# The Role of Paternal Involvement on Behavioral Sensitive Responses and Neurobiological Activations in Fathers: A Systematic Review

**DOI:** 10.3389/fnbeh.2022.820884

**Published:** 2022-03-09

**Authors:** Michele Giannotti, Micol Gemignani, Paola Rigo, Paola Venuti, Simona De Falco

**Affiliations:** ^1^Department of Psychology and Cognitive Sciences, University of Trento, Trento, Italy; ^2^Department of Developmental Psychology and Socialisation, University of Padua, Padua, Italy

**Keywords:** father, paternal involvement, paternal sensitivity, parental brain, paternal behavior, fatherhood, neurobiology

## Abstract

As fathering research has flourished, a growing body of studies has focused on behavioral and neurobiological mechanisms, respectively associated with caregiving sensitivity and responsiveness to infant stimuli. However, the association between these aspects and the key concept of paternal involvement in childcare (i.e., contribution in infant care in terms of time, availability, and responsibility) has been poorly investigated. The current work aims to systematically review the role of involvement in childcare on both neural activations and sensitive behaviors in fathers by examining (a) how paternal involvement has been measured and (b) whether paternal involvement has been associated with neurobiological activation and behavioral sensitive responses. Inclusion criteria were peer-reviewed quantitative studies, concerning fathers responding to infant stimuli at neurobiological or behavioral level, and including a quantitative measurement of paternal involvement in childcare. A quality rating for each study has been performed based on the measurements adopted to assess paternal involvement. Of 2,529 articles, 27 studies were included. According to our quality rating, 10 out of 27 studies included fairly good-standard measures for measuring paternal involvement, whereas 17 studies used good-standard measures. In addition, 11 studies provided details of paternal involvement in the context of neurobiological responses to infant stimuli, whereas 16 addressed paternal sensitive behaviors. Overall, only 8 studies reported relevant findings about the relationship between paternal involvement and neurobiological responses or sensitive behaviors in fathers. The present study is the first systematically evaluating the scope of paternal involvement in the field of Paternal Brain and fathers' sensitive responsiveness research. When high-standard measures are used, paternal involvement seems to play a significant role in modulating both the hormonal and the neural pathways associated with paternal behaviors. Remarkably, the role of paternal engagement may underpin an adaptive nurturance that is not dependent on pregnancy and childbirth but on caregiving experience. A promising positive link between paternal involvement and behavioral sensitivity may be expected in further studies, which will need to corroborate our conclusion by adopting detailed and appropriate measures assessing paternal involvement. As a future line of research, the inclusion of gay fathers may be beneficial for the field.

## Introduction

Due to the contemporary socio-cultural changes, fathers have been increasingly involved in child rearing activities, providing more time, care, and emotional support to their offspring (Schoppe-Sullivan and Fagan, 2020). Accordingly, over the past years a growing body of parenting research has progressively focused on the study of paternal role and its influence on the healthy development of infants (Ramchandani et al., 2011; Lamb and Lewis, 2013; Leidy et al., 2013). In this regard, several studies have addressed multiple components of fathering, including neurobiological, psychological, and behavioral mechanisms that support adequate parental caregiving. However, the association between neurobiological activations to infant stimuli, paternal behavioral sensitivity and the key concept of paternal involvement in caring for their own children has been poorly investigated. Nonetheless, fathers' involvement in childcare has proved of paramount importance within family contexts, with research demonstrating its positive impact on both mother and child's health outcomes (Yargawa and Leonardi-Bee, 2015; Taylor et al., 2020). Unlike previous remarkable works in the field (e.g., Rilling and Mascaro, 2017; Storey et al., 2020) the present systematic review will uniquely go through recent research on neurobiological and behavioral aspects of fatherhood through the lenses of paternal involvement. In fact, no research to date has provided an overview of paternal neurobiology by linking differential neural and hormonal pathways to individual variations in paternal involvement.

### Behavioral Studies on Paternal Sensitivity and Responsiveness

According to the Attachment Theory early conceptualization (Bowlby, 1969, 1982), parental sensitivity is defined as the ability to recognize, interpret, and provide adequate and prompt responses to children's cues (Ainsworth et al., 1978). Thus, it involves awareness of the infant/child emotional and mental states, emotional support, engagement in a mutually rewarding interaction and appropriateness based on the child developmental capacities (Nicholls and Kirkland, 1996). Behavioral sensitivity, as a broad concept, has been traditionally assessed by coding parental behaviors in the context of parent-child dyadic interactions. The study of parental sensitivity and responsiveness focused predominantly on mothers, as they have been historically considered as the child's primary caregiver. Nevertheless, due to the increased involvement of fathers in childcare (Craig and Mullan, 2010), a growing number of studies have examined paternal sensitivity and its contribution to child development. In this regard, several studies highlighted that sensitive fathers respond accurately to children's signals and needs, showing an appropriate attunement and pattern of interaction in different contexts (Towe-Goodman et al., 2014; Branger et al., 2019). Empirical evidence documented the association between paternal sensitivity and various child's outcomes such as cognitive functioning, emotion regulation, externalizing behaviors, and attachment security (Lucassen et al., 2011; Rodrigues et al., 2021). Compared to mothers, it has been observed that fathers frequently displayed lower levels of parental sensitivity (Hallers-Haalboom et al., 2014), even in cases in which their children showed equal attachment security and responsiveness to both parents (Kochanska and Aksan, 2004; Lickenbrock and Braungart-Rieker, 2015). This gender difference can be explained by different reasons, including the methods used for the assessment, which have been originally developed for mothers (Mesman and Emmen, 2013). In addition, father-child interactions are characterized by specific features which should be taken into account to better understand the role of parental sensitivity and its relation with father-infant attachment and child's developmental outcomes. In fact, it has been suggested that fathers are more focused on stimulation and explorative play, encouraging risk-taking more frequently than mothers in the interaction with their children (Lucassen et al., 2011; Cabrera et al., 2014; Olsavsky et al., 2020). Moreover, several studies documented that physical play, particularly rough-and-tumble behaviors, is a common form of dyadic interaction between fathers and their children (Amodia-Bidakowska et al., 2020). However, this peculiarity of the father-child relationship does not properly reflect paternal experiences in the context of contemporary families (Cabrera et al., 2000), as in the case of single gay and heterosexual fathers. In fact, another relevant factor that may significantly contribute to paternal sensitive behaviors is the degree of father involvement in childcare (Cabrera and Tamis-LeMonda, 2013). In this regard, a longitudinal study (Brown et al., 2012) has shown the association between paternal involvement, paternal sensitivity, and child attachment. Nevertheless, very little is known about how paternal involvement contributes to fathering behaviors in terms of behavioral sensitivity and responsiveness to children's signals.

### Neurobiology of the Paternal Brain

In line with the Parental Brain Model (Swain, 2011), it is notable that key brain circuits are activated when parents are exposed to visual and auditory infant stimuli. To date, functional adaptations in fathers' brains have been demonstrated to come along with hormonal changes supporting caregiving behaviors (Storey et al., 2020). According to relevant evolutionary perspectives (Mascaro et al., 2013; Wingfield, 2017), a wide range of studies have shown a general decrease of Testosterone levels for fathers (Mascaro et al., 2014), with this being associated with an enhanced quality of nurturant behaviors (Fleming et al., 2002; Weisman et al., 2013; Gordon et al., 2017; Roellke et al., 2019). Conversely, high levels of Oxytocin, Vasopressin, and Prolactin have been generally linked to a greater amount of paternal synchrony and responsiveness (Gordon et al., 2010; Atzil et al., 2012). In addition, changes in Cortisol levels have been reported for fathers (Kuo et al., 2018). Moving to the neural activations, multiple studies have shown that fathers usually recruit neural systems tapping into sensory information processing and integration, motivation, and empathy in response to visual or auditory infant stimuli (Mascaro et al., 2014; Kim et al., 2015; Li et al., 2018). For instance, Mascaro et al. (2013) found that cry sounds robustly activated brain regions including the bilateral Inferior Frontal Gyrus and extending into the Anterior Insula. In response to pictures of children, fathers showed stronger activations than non-fathers within regions important for face emotion processing (e.g Caudal Middle Frontal Gyrus), mentalizing (e.g., Temporo-Parietal Junction), and reward processing (e.g., Medial Orbitofrontal cortex) (Mascaro et al., 2014). In line with the hypothesis that neural changes may allow for better caregiving of the offspring, fathers also showed great activations in reward- and attachment-related brain regions (e.g., left Globus Pallidus, medial Orbitofrontal Cortex, left Hippocampus, bilateral inferior Frontal Gyrus, Anterior Insula) in response to their own infants (Wittfoth-Schardt et al., 2012). Trying to provide an accurate idea of the Parental Brain in fathers, Provenzi et al. (2021) consistently reported the activation of three brain networks when fathers respond to infant cues, respectively linked to mentalization (e.g., Superior Temporal Sulcus, Medial Prefrontal Cortex), embodied simulation (e.g., Anterior Insula, Middle and Lateral Superior Frontal Gyrus, Ventral Anterior Cingulate Cortex), and emotion regulation processes (e.g., Inferior Frontal Gyrus, Orbitofrontal Cortex). In addition to these, the activation of subcortical structures (e.g., Caudate, Putamen, Globus Pallidus, thalamus, Substantia Nigra, Amygdala) have been reported in fathers. Providing some remarkable differences between mothers and fathers, whilst fathers' neural responses to infant cues seem to rely more on socio-cognitive networks, mothers' activations mainly include limbic regions (Atzil et al., 2012; Rajhans et al., 2019). Even though this evidence is not called into question and some Parental Brain characteristics may be actually hard-wired and sex-specific, it should be acknowledged that most research in the field has failed to take the variability related to parental involvement into consideration (Provenzi et al., 2021). Stressing the importance of caregiving experience, it may also be the case that fathers' neuroendocrine system is responsive to committed parenting (Weisman et al., 2014). In this theoretical framework, it would be advisable to investigate the relationship between paternal involvement and neurobiological responses to infant cues, by focusing on functional brain activations and hormonal regulations when fathers respond to infant stimuli.

### Paternal Involvement

Broadly speaking, paternal involvement consists of the quantity of time fathers positively engage with—and are available to—their child, and the load of responsibilities they decide to take on for their child's welfare (Lamb et al., 1985; Brown et al., 2012). Notably, existing research has repeatedly demonstrated a link between fathers' involvement and children's developmental outcomes, with less paternal engagement being associated with poorer children psychological wellbeing, social and adaptive behaviors, intellectual functioning, academic achievements, language development, but an higher incidence of externalizing behaviors (Aldus and Mulligan, 2002; Tamis-LeMonda and Cabrera, 2002; Lamb, 2010; Jia et al., 2012). As an instance, encouraging paternal involvement in the child's upbringing has proved to bring moderate to high gains to children in terms of cognitive functioning (Cano et al., 2019). Indeed, the effect of paternal co-parenting has been demonstrated to positively impact the pediatric outcomes (Tikotzky et al., 2011) even in cases in which a chronic illness occurs (Taylor et al., 2020). Furthermore, paternal engagement with childcare improved family contexts and had downstream positive effects on the child in terms of cognitive development (Pleck, 2010; Cano et al., 2019). In terms of marital relationship, male involvement has proved to foster mothers' health outcomes and positive behaviors both prenatally and postnatally (Giurgescu and Templin, 2015; Yargawa and Leonardi-Bee, 2015; Kortsmit et al., 2020). Moreover, paternal engagement with their own child was revealed to have a buffering effect by reducing the adverse long-term effects of maternal depression on later child's internalizing problems (Mezulis et al., 2004). Despite its relevance, it is worth noting that different worldwide policies and cultural contexts may have had a direct and indirect effect on paternal involvement. Indeed, the time fathers may allocate on households and the gender division of labor have strictly depended on local national policies (Craig and Mullan, 2010). Along with this, socio-cultural assets have potentially had an influence on fathers' personal norms and expectations regarding its role as caregiver, thus affecting the personal child-rearing attitudes (Bakermans-Kranenburg et al., 2019). Those issues being mentioned, findings so far have pointed to an encouraging direction, according to which fathers' involvement plays a positive role in promoting the overall child and family's well being. However, one limitation of this research is that different conceptualizations of involvement have led to a great heterogeneity in terms of measures, with some authors considering only partial components of that core construct or collapsing heterogeneous aspects into a limited measure (Chen and Zhu, 2017). If endorsed by researchers in this field of study, a more appropriate attitude regarding methodological and theoretical issues could actually improve the generalization of results concerning the positive impact of fathers' engagement within family contexts. In addition, little research so far has focused on the inter-relationships between paternal involvement and the key constructs of Paternal Brain and behavioral sensitivity. As aforementioned, putting in relation these factors by using a fine methodology could definitely provide new insights into the importance of fathers' different characteristics for the entire family unit.

### Objectives

The general purpose of this study is to systematically review literature regarding the contribution of father involvement on paternal sensitive behaviors and neurobiological responses to infant stimuli, thereby including both neurobiological and behavioral studies. Specifically, the current study aims: (a) to examine the type and quality of the measures used to assess father involvement; (b) to explore the link between the degree of caregiving involvement in fathers and the level of paternal sensitivity and responsiveness both at behavioral and neurobiological level.

## Method

The current review has been conducted following the methodological guidelines recommended by the PRISMA (Preferred Reporting Items for Systematic Reviews and MetaAnalysis) (Moher et al., 2009). After framing the research questions, we developed the search strategy and the data collection method, and we defined the inclusion and exclusion criteria in relation to the study aims. Eventually, we assessed the risk of bias of each study included. The search strategy and the outcomes, including data selection, synthesis, presentation and interpretation are fully described below.

### Search Strategy

We comprehensively searched the literature on neurobiological responses and sensitive behaviors through two electronic databases (PsycInfo, PubMed) until March 2021. In addition, a search of the relevant articles and the reference lists has been conducted manually. To this aim, the initial records were entered into electronic databases to identify papers citing these articles. The search strategy was undertaken by two independent researchers considering only studies published in English after 2000. In this initial stage, no specific limits were applied with respect to publications status or the study designs. Search terms were formulated based on an interpretation of the Population/Problem of interest, phenomenon of Interest and Context (PICo) (Joanna Briggs Institute, 2014).

The following search terms were used: (Paternal OR Father) AND (Sensitivity OR Responsiveness) AND (Brain OR Hormones OR Neural OR Father-child interaction OR Child care). Notably, the heterogeneous conceptualizations of paternal involvement have resulted in the concept being present neither in the MeSH nor in the APA Thesaurus of Psychological Index Terms. For this reason, we have not included a keyword related to paternal involvement in the initial search, but we went through all the studies in order to identify all the different ways to assess the core concept across research.

### Eligibility Criteria

The following inclusion criteria have been met in this review: (a) reported quantitative data; (b) published in peer-reviewed journals; (c) included the assessment of paternal sensitivity behaviors (i.e., father-child interactions or implicit responsiveness in behavioral tasks) or neurobiological responses to child stimuli (i.e., functional neural correlates, hormonal responses); (d) included fathers in the study sample (i.e., only fathers or both mothers and fathers); (e) reported data on humans; (f) included a quantitative measurement of paternal involvement in childcare.

On the other hand, exclusion criteria were the following: (a) focused on parents with a certified psychiatric diagnosis or children with atypical development (e.g., neurodevelopmental disorders); (b) included only adolescents rather than infants or children in the study sample; (c) based exclusively on self-reported measurements of parental sensitivity and responsiveness.

### Study Selection

**Figure 1:**
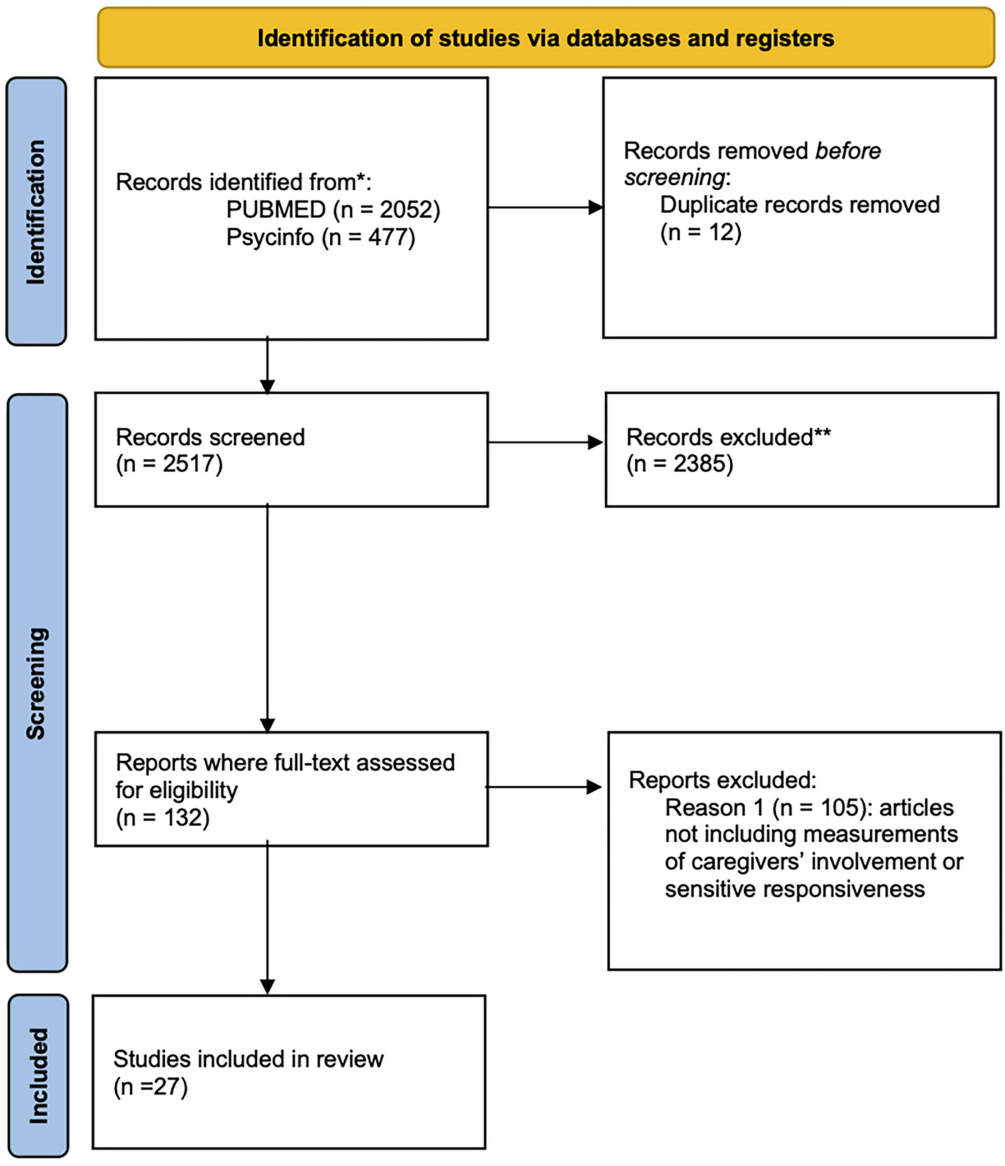
PRISMA flow diagram. ^*^Consider, if feasible to do so, reporting the number of records identified from each database or register searched (rather than the total number across all databases/registers). ^**^If automation tools were used, indicate how many records were excluded by a human and how many were excluded by automation tools. From: Page et al. (2021).

The first screening of the studies was conducted according to the specific inclusion criteria based on abstract and title, after having checked for duplicates by using Zotero 5.0.96.2. Relying on the full text, we then rejected articles that met the exclusion criteria. In particular, a large number of neurobiological studies reported data on animal studies, or included only mothers, whereas behavioral studies often focused on parents' psychopathology or children's atypical development. In general, the majority of studies on paternal sensitivity and responsiveness did not include a quantitative measurement of parental involvement, which constituted the primary reason for exclusion. The study selection process is fully summarized using the PRISMA flow diagram (Figure 1).

### Data Extraction and Management

We developed a standardized spreadsheet including all the studies meeting the eligibility criteria. The first independent reviewer (MGI) collected data from the selected articles and the second reviewer (MGE) checked the extracted data. Discrepancies between the two independent reviewers (MGI and MGE) were discussed and solved and a third author was involved in the decision if no agreement was reached. Specifically, the data extracted included: (a) study characteristics (i.e., authors, year of publication, design); (b) sample characteristics (i.e., N, sample type, country, father and child age, socioeconomic status, developmental period,); (c) measures/methods used for the assessment of paternal sensitivity/responsiveness; (d) constructs related to parental sensitivity/responsiveness, (e) measures of paternal involvement in childcare; (f) outcomes of paternal sensitivity/responsiveness associated with paternal involvement of fathers.

### Assessment of Risk of Bias

Due to the large heterogeneity of the characteristics of the studies included the risk of bias of each study was assessed using the Mixed Methods Appraisal Tool 2018 (MMAT) (Hong et al., 2018). This checklist is designed to appraise the methodological quality of five different types of empirical studies: qualitative research, randomized controlled trials, non-randomized studies, quantitative descriptive studies, and mixed methods studies. It consists of two initial screening questions on clarity of research questions and its relation with data collection. In the second section, MMAT includes five methodological quality criteria questions for each category of study type. Once the study type has been selected, two independent reviewers (MGI and MGE) rated the seven quality criteria for each included study using one of the possible responses (“Yes,” “No,” “Can't tell,” “Comments”). Overall, the reviewers reached 93.75% of agreement for the rating. Disagreements based on different interpretations of the study characteristics were solved through the supervision of the third author. The quality appraisal of the included studies is reported in Supplementary Materials for neurobiological (Supplementary Table 1) and behavioral studies (Supplementary Table 2).

### Data Synthesis

According to the aims of this systematic review, results were synthesized as follows. Firstly, we reported the characteristics of the selected studies (Table 1), including the authors, title, country, study type, study design, sample, child developmental period, measure of paternal sensitivity behaviors or neurobiological responsiveness to infant cues, measure of paternal involvement, and the quality of rating. Therefore, the assessment of paternal involvement used in the included studies was described systematically, and two independent reviewers (MGI and MGE) provided a binary evaluation by assigning a rating in terms of “good” or “fairly good” for each measurement of paternal involvement. Additionally, we specified whether the role of involvement was addressed in the research hypotheses, and whether results involving fathers' involvement were presented.

**Table 1:** Characteristics of the selected studies.

**Authors**	**Title**	**Country**	**Study type**	**Study design**	**Sample**	**Developmental stage**	**Neurobiological or sensitivity measure**	**Paternal involvement measure**	**Quality rating**	**Is the role of involvement addressed in the research hypotheses?**	**Are there any results for involvement?**
Boechler et al. (2003)	Father-child teaching interactions: the relationship to father involvement in caregiving	Canada	Behavioral study	Quantitative non-randomized study	Fathers (*N* = 110)	Perinatal	Nursing Child Assessment Teaching Scale (NCATS)	One item on how often the father had sole responsibility for the child in the previous week	+	Yes	No
Brown et al. (2018)	Associations between father involvement and father–child attachment security: Variations based on timing and type of involvement	USA	Behavioral study	Quantitative non-randomized study	Fathers (*N* = 80)	Postnatal	15-min parent-child interactions	Interaction/ Accessibility Time Diary interview protocol	++	Yes	No
Brown et al. (2012)	Father involvement, paternal sensitivity, and father-child attachment security in the first three years	USA	Behavioral study	Quantitative non-randomized study	Fathers (*N* = 115, 71 at both timepoints)	Postnatal	10-minute competing demands task coded using a global 5-point (1 = highly insensitive, 5 = highly sensitive) rating scale	Adapted version of the Parental Responsibility Scale	++	Yes	Yes
Carone et al. (2020)	Gay and heterosexual single father families created by surrogacy: father–child relationships, parenting quality, and children's psychological adjustment	Italy	Behavioral study	Quantitative non-randomized study	Fathers (*N* = 35 gay single father, *N* = 30 heterosexual single father, *N* = 45 gay two-father families, *N* = 45 heterosexual two-parent families)	Postnatal	Coding of Attachment-Related Parenting (CARP)	Interview including code on parental investment/ involvement, describing the father's belief in the importance of being a parent and clear commitment to parenting	++	No	No
Feldman (2000)	Parents' convergence on sharing and marital satisfaction, father involvement, and parent–child relationship at the transition to parenthood	Israel	Behavioral study	Quantitative non-randomized study	60 Israeli couples	Postnatal	Three videotaped interactions (mother–child, father–child, and a triadic family interaction) coded using a system developed by the author	Self-report questionnaires assessing how parents share household and childcare responsibilities, time spent with the infant and range of parenting activities	++	Yes	Yes
Feugé et al. (2020)	Adoptive gay fathers' sensitivity and child attachment and behavior problems	Canada	Behavioral study	Quantitative non-randomized study	Homosexual fathers (*N* = 68)	Postnatal	Maternal Behavior Q-Sort short form (MBQS)	Self-assessment on a parental involvement scale	++	No	No
Fuertes et al. (2016)	The effects of parental sensitivity and involvement in caregiving on mother–infant and father–infant attachment in a Portuguese sample	Portugal	Behavioral study	Quantitative non-randomized study	Mothers (*N* = 82) and fathers (*N* = 82)	Perinatal- postnatal ^*f*^	CARE-Index	Parents' Responsibility Scale-Portuguese version	++	Yes	No
Grossmann et al. (2002)	The uniqueness of the child–father attachment relationship: fathers' sensitive and challenging play as a pivotal variable in a 16-year longitudinal study	Germany	Behavioral study	Quantitative non-randomized study	Mothers (*N* = 49) and fathers (*N* = 49)	Perinatal-postnatal	Ainsworth's scale of maternal sensitivity to the infant's communication; Sensitive and Challenging Interactive Play Scale (SCIP Scale)	Maternal report of paternal involvement and father-child observation	+	No	Yes
Kazura (2000)	Fathers' qualitative and quantitative involvement: an investigation of attachment, play, and social interactions	USA	Behavioral study	Quantitative non-randomized study	Mothers (*N* = 27), fathers (*N* = 27)	Postnatal	Belsky and Most's categories of functional and symbolic play, Prelinguistic Infant-Parent Communicative Interaction Code	Parent-Child Caregiving Questionnaire	++	Yes	No
Knauer et al. (2019)	Parenting quality at two developmental periods in early childhood and their association with child development	Mexico	Behavioral study	Quantitative non-randomized study	Mothers and fathers (605 famiglie)	Perinatal- postnatal	The Home Observation for Measurement of the Environment (HOME) Inventory	The Home Observation for Measurement of the Environment (HOME) Inventory	+	Yes	No
Laflamme et al. (2002)	A comparison of fathers' and mothers' involvement in childcare and stimulation behaviors during free-play with their infants at 9 and 15 months	Canada	Behavioral study	Quantitative non-randomized study	Mothers (*N* = 87) and fathers (*N* = 87)	Perinatal- postnatal	Free-play observation	An adapted version of the Parental Responsibility Scale (PRS) and a Daily journal	++	Yes	No
Lewis et al. (2009)	A comparison of father–infant interaction between primary and non-primary care giving fathers	England	Behavioral study	Quantitative non-randomized study	Fathers (25 primary caregivers, 75 non-primary caregivers)	Perinatale	Global sensitivity scale of Ainsworth, facilitation scale	Maternal report of paternal involvement in daily care (i.e. h/week fathers take sole responsibility and paternal decision making in the couple regarding the child)	++	Yes	No
Lundy (2002)	Paternal socio-psychological factors and infant attachment: The mediating role of synchrony in father–infant interactions	USA	Behavioral study	Quantitative non-randomized study	Mothers (*N* = 15) and fathers (*N* = 15)	Perinatal	A modified version of synchronous parent–infant exchanges	The amount of time regularly spent interacting (i.e., one-on-one) with the infant, and percentage of father–infant vs. mother–infant care	+	Yes	No
Malmberg et al. (2016)	The influence of mothers' and fathers' sensitivity in the first year of life on children's cognitive outcomes at 18 and 36 months	England	Behavioral study	Quantitative non-randomized study	Mothers (*N* = 97) and fathers (*N* = 97)	Perinatal- postnatal	Global sensitivity scale of Ainsworth, facilitation scale	Primary caregiver defined as a minimum of 20 waking hours a week of sole child-care	++	No	No
Malmberg et al. (2007)	Parent–infant interaction: A growth model approach	England	Behavioral study	Quantitative non-randomized study	Mothers (*N* = 1,077), primary caregiver mothers (*N* = 25), non- primary caregiver fathers (*N* = 75)	Perinatal	Global sensitivity scale of Ainsworth, facilitation scale	Primary caregiver defined as a minimum of 20 waking hours a week of sole child-care	++	No	No
National Institute of Child, Health, and Human Development Early Child Care Research Network (2000)	Factors associated with fathers' caregiving activities and sensitivity with young children	USA	Behavioral study	Quantitative non-randomized study	Fathers (*N* = 585, *N* = 278 at 6 months, *N* = 184 at 36 months)	Perinatal-postnatal	4 point rating scale (6 months), 7 point rating scale (35 months)	Questionnaire (15 items) describing parents' responsibilities for caregiving activities at 6,15, 24, and 36 months of child age	++	Yes	No
Abraham et al. (2014)	Father's brain is sensitive to childcare experiences	Israel	Neurobiological study	Quantitative non-randomized study	Mothers and fathers (eterosexual primary caregiver mothers = 20, eterosexual secondary caregiver fathers = 21, homosexual primary caregiver fathers = 48)	Perinatal/ postnatal^d^	Neural response to infant stimuli (fMRI)	Structured interview to determine the parent's caregiving responsibilities and primary caregiving role	++	Yes	Yes
Feldman et al. (2010)	Natural variations in maternal and paternal care are associated with systematic changes in oxytocin following parent–infant contact	Israel	Neurobiological study	Quantitative non-randomized study	Mothers (*N* = 71) and fathers (*N* = 41)	Perinatal	Hormonal response after interaction	Two items related to house-care responsibilities and childcare responsibilities	+	No	No
Gettler et al. (2013)	Progesterone and estrogen responsiveness to father-toddler interaction	Philippines	Neurobiological study	Quantitative non-randomized study	Fathers (*N* = 44)	Postnatal	Hormonal response after interaction	An item based on routinely playing with children	+	Yes	No
Gettler et al. (2011)	Short-term changes in fathers' hormones during father–child play: Impacts of paternal attitudes and experience	Philippines	Neurobiological study	Quantitative non-randomized study	Fathers (*N* = 45)	Postnatal	Hormonal response after interaction	Items on caregiving behaviors including feeding children, playing, bathing children, reading to children, and walking children to school	++	Yes	Yes
Kuo et al. (2018)	Fathers' cortisol and testosterone in the days around infants' births predict later paternal involvement	USA	Neurobiological study	Quantitative non-randomized study	Fathers (*N* = 298)	Perinatal^c^	Hormonal response after interaction	Childcare Activities Scale	++^a^	Yes	Yes
Kuo et al. (2016)	Individual variation in fathers' testosterone reactivity to infant distress predicts parenting behaviors with their 1-year-old infants	USA	Neurobiological study	Quantitative non-randomized study	Fathers (*N* = 175)	Perinatal	Hormonal response after interaction	Joint couple interview to assess the division of labor	+^b^	Yes	No
Mascaro et al. (2014)	Behavioral and genetic correlates of the neural response to infant crying among human fathers	USA	Neurobiological study	Quantitative non-randomized study	Fathers (*N* = 36)	Postnatal	Neural response to infant stimuli (fMRI)	Parental Responsibility Scale	++	Yes	Yes
Mascaro et al. (2013)	Testicular volume is inversely correlated with nurturing-related brain activity in human fathers.	USA	Neurobiological study	Quantitative non-randomized study	Fathers (*N* = 70)	Postnatal^d^	Neural response to infant stimuli (fMRI)	Parental Responsibility Scale	++	Yes	Yes
Nishitani et al. (2017)	Genetic variants in oxytocin receptor and arginine-vasopressin receptor 1A are associated with the neural correlates of maternal and paternal affection toward their child	Japan	Neurobiological study	Quantitative non-randomized study	Mothers (*N* = 43) and fathers (*N* = 41)	Perinatal/ postnatal	Neural response to infant stimuli (fNIRS)	Two items related to childcare responsibilities	+	No	No
Waller et al. (2015)	Attachment representation modulates oxytocin effects on the processing of own-child faces in fathers	Germany	Neurobiological study	Quantitative randomized controlled trials	Fathers (*N* = 32)	Postnatal	Neural response to infant stimuli (EEG)	Question based on the quantity of time spent with their child per week	+	No	No
Wittfoth-Schardt et al. (2012)	Oxytocin modulates neural reactivity to children's faces as a function of social salience	Germany	Neurobiological study	Quantitative randomized controlled trials	Fathers (*N* = 21)	Postnatal	Neural response to infant stimuli (fMRI)	Question based on the quantity of time spent with the child per week	+	No	No

a *++: involvement measure classified as “good”*.

b *+: involvement measure classified as “fairly good”*.

c *Perinatal stage: from childbirth to 1 year of age of children*.

d *Perinatal/postnatal: children included in the studies are in both the developmental stages*.

e *Postnatal: beyond 1 year of age of children*.

f *Perinatal-postnatal: longitudinal studies from perinatal to postnatal period*.

## Results

At the first stage, the database search generated 2,529 records, from which 12 duplicates were removed before the screening process. Of 2,517 potential records identified in the initial screening, 2,385 were excluded during the screening of titles and abstracts. Thus, 132 were assessed for full-text eligibility and 27 met study inclusion criteria. No further eligible studies were identified from reference list screening. PRISMA flow diagram is presented in Figure 1.

### Study Characteristics

Of the 27 included studies, 11 (40.7%) focused on neurobiological aspects, and 16 (59.3%) on paternal sensitive and responsive behaviors. In the context of neurobiological studies, parental responses has been measured through hormonal responses after parent-child interactions (*n* = 5; 18.5%), or neural activations to infant stimuli (i.e., faces, cry) using fMRI, EEG or fNIRS (*n* = 6; 22.2%). All the included behavioral studies assessed paternal sensitive behaviors in the context of parent–child interactions using observational methods based on different standardized coding. The characteristics of the studies are summarized in Table 1. Almost all the studies were based on non-quantitative randomized design (*n* = 25; 92.6%) and only 2 studies (7.4%) were randomized control trials. With respect to the sample, 12 studies (44.4%) involved both mothers and fathers and 15 (55.6%) included only men. Among these, three studies (11.5%) also included homosexual fathers. Of the 27 studies included, 7 (25.9%) have been conducted during perinatal period (i.e., the period up to 12 months after childbirth) and 12 (44.4%) postnatally. Six studies (22.2%) were longitudinal, starting from the perinatal period and including follow-up assessments afterwards. Only 2 out of 27 studies (7.4%) included children both below and above the age of 12 months. Seven neurobiological studies (25.9%) and 11 behavioral studies (40.7%) included the variable of paternal involvement in their research questions.

### Measurement of Paternal Involvement (Quality Rating)

We evaluated the measures used for the assessment of paternal involvement in terms of “good” or “fairly good” depending on how thorough and detailed multiple aspects of paternal involvement have been considered (Table 1). All the articles included in this review have been considered in the Discussion section, independently whether they presented “good” or “fairly good” measures for assessing paternal involvement. According to the quality rating, 10 out of 27 studies included “fairly good” measures for assessing paternal involvement, with 6 measuring paternal neurobiological responses to infant cues and 4 involving behavioral aspects of parental sensitivity. The criteria we considered for measures to be classified as “fairly good” were: (a) being made of one item or few items; (b) being rated or scored with poor methodological clarity or soundness; (c) being generated from a set of measures not specifically developed for the assessment of paternal involvement.

On the other hand, we reported that the remaining 17 studies included in this review used “good” measures for evaluating paternal involvement, with 5 assessing parental responses in the context of neurobiology and 12 studies involving behavioral and parent-child interactions. The types of measurements we included in this subgroup were: (a) well-established questionnaires for measuring parental involvement; (b) appropriate structured or well-founded interviews or daily journals; (c) *ad-hoc* detailed sets of questions or specific instruments purposefully developed for the study. The studies fulfilling the first criterion predominantly adopted the Childcare Activity Scale (Cronenwett et al., 1988), the Parental Responsibility Scale (McBride and Mills, 1993), or the Parental Engagement Questionnaire (Dubeau et al., 2009) as *ad-hoc* instruments to measure paternal involvement. Concerning the second criterion, a structured interview to determine the caregiving responsibilities and primary caregiving role of parents was used by Abraham et al. (2014), and it was based on The Early Childhood Longitudinal Study, Birth Cohort Father's Questionnaire (http://nces.ed.gov/ecls/birth.asp) and the Father's Daily Routines Questionnaire (Goldberg and Easterbrooks, 1984).

Differently, a widely used interview in the father involvement literature, that is the Interaction/Accessibility Time Diary interview protocol, was utilized by Brown et al. (2012, 2018). As another example, Laflamme et al. (2002) asked parents to record the time spent in specific activities by means of a daily journal. For the third criterion, a fine set of different but detailed questions was utilized by Lewis et al. (2009) and Gettler et al. (2011). Moreover, Feldman (2000) extensively assessed five determinants of father involvement by using self-reported questionnaires. Eventually, Kazura (2000) specifically developed an instrument to address the level of involvement and division of labor between the parents caring for children.

### Associations Between Paternal Involvement and Sensitive Behavioral Responses

Of the 16 studies addressing behavioral paternal sensitive responsiveness, 12 used “good” measures of paternal involvement. On the other hand, among the 5 studies including a “fairly good” measure, only one (i.e., Grossmann et al., 2002) found a significant effect of paternal involvement on sensitive behavioral responses in fathers. Specifically, the composite caregiving index assessed during the first year of the infant's life, also including the mother-reported level of paternal caregiving involvement, significantly predicted father sensitive play 24 months after childbirth. However, further analyses have shown that this effect was explained exclusively by the presence of the father at birth rather than the overall degree of caregiving involvement during the first year. Among the 12 studies using “good” measures for assessing paternal involvement, only two (Feldman, 2000; Brown et al., 2012) reported a significant association between paternal involvement and sensitive behaviors in fathers. In the first mentioned study, paternal involvement, sensitivity, and child attachment have been assessed at two timepoints (i.e., 13 months, 3 years). A significant negative relationship between paternal involvement and sensitivity was found at 3 years. Nevertheless, no associations emerged between involvement and sensitivity at 13 months. As far as the second study is concerned (Feldman, 2000), sensitive fathering proved related to four characteristics of father involvement, such as sharing of household and childcare responsibilities, the amount of time fathers spent with the child on weekends (but not during the week), and the range of childcare activities fathers performed. In addition to these, other studies reported no significant association between paternal involvement measured with quantitative variables and parental sensitivity (Kazura, 2000; Laflamme et al., 2002; Malmberg et al., 2016).

### Associations Between Involvement and Paternal Neurobiological Responses

Of 2,529 articles, 11 studies provided details of paternal involvement in the context of neurobiological responses to infant stimuli. As mentioned before, 6 out of 11 studies included “fairly” good measures for assessing paternal involvement, whereas 5 studies used “good” measures. Of note, those studies classified as adopting “fairly good” measures failed to find significant relationships between paternal involvement and neurobiological responses to infant cues. On the other hand, a relevant effect of paternal involvement has been highlighted in those studies measuring the construct more thoroughly. Going into details, paternal reactivity Cortisol after interactions with newborns has been found predictive of greater paternal involvement in childcare and play (Kuo et al., 2018). Specifically, fathers whose Cortisol levels increased significantly while holding their infants reported greater postpartum involvement in indirect care and play. Differently, a downregulation in the Prolactin levels while interacting with their own toddlers has been found in those fathers spending more time in daily caregiving when compared to fathers less involved in childcare (Gettler et al., 2011). At the neural level, greater activity in the Ventral Tegmental Area in response to pictures of their own children was associated with higher paternal involvement (Mascaro et al., 2013). In addition, a moderate level of Anterior Insula activity in response to infant cry was related to high levels of instrumental support of fathers, as being reported by mothers (Mascaro et al., 2014). Abraham et al. (2014) displayed that fathers' direct caregiving experiences correlated with the Amygdala and Superior Temporal Sulcus connectivity in response to self-infant interactions. Additionally, more involved fathers (i.e., primary-caregiving fathers) showed a high Amygdala activation as primary-caregiving mothers, and a high Superior Temporal Sulcus activation as secondary-caregiving fathers.

## Discussion

The main aim of this work was to elucidate the role of paternal involvement in sensitive behaviors of father and neurobiological responses to infant cues, by examining both neurobiological and behavioral empirical studies. Overall, only 8 studies reported results related to paternal involvement, and the majority of which (*n* = 5; Gettler et al., 2011; Mascaro et al., 2013, 2014; Abraham et al., 2014; Kuo et al., 2018) investigated neurophysiological mechanisms underlying paternal responses to infant cues. Results from these studies confirmed the presence of a significant link between hormonal changes (i.e., Cortisol, Prolactin), neural activations (i.e., Anterior Insula, Ventral Tegmental Area) and the degree of parental involvement. With respect to behavioral research, little but very promising evidence emerged for the association between paternal involvement and sensitive behaviors in the context of parent–child interactions (Feldman, 2000).

### Study Characteristics

In this systematic review, the number of neurobiological (40.7%) and behavioral studies (59.3%) addressing paternal involvement and sensitive behaviors or neurobiological responses to infant cues were fairly balanced. Whilst 15 studies included a sample of only fathers, 12 involved both parents, allowing a more comprehensive assessment of the processes which characterize paternal involvement in the light of the couple and family functioning. It is noteworthy that only two studies included homosexual primary caregiving fathers (Abraham et al., 2014; Feugé et al., 2020), and another study considered both gay families and single fathers in the targeted sample (Carone et al., 2020). In this regard, the inclusion of new family forms in the investigation of paternal involvement, especially referring to parents genetically unrelated to children, might provide some fascinating insights into the effects of engaging with active childcare. Thus, by ruling out the impact of those mechanisms strictly driven by nature (i.e., pregnancy), families of gay fathers might offer the unique opportunity to assess the developmental consequences of paternal involvement when fathers are the primary caregivers (Carone et al., 2020). Moreover, none of the studies included in this review have been conducted in the prenatal period. This evidence raises concern on how the measurement inconsistencies at that developmental stage, together with the lack of a clear conceptual framework, could result in an overall paucity of data. Although the physical absence of babies in the prenatal period might cause problems in finding reliable and sensitive measures for a comprehensive assessment, a growing body of research is showing that high prenatal attachment is associated with positive parenting behaviors in fathers after childbirth (Lindstedt et al., 2020). By clarifying some missing gaps in the theoretical framework, we therefore suggest that the core aspects of paternal involvement in the prenatal period should be accurately identified (Chen and Zhu, 2017). Moving forward to other study characteristics, namely the country of origin or the socio-economic status, it is essential to consider that the heterogeneity of the targeted populations and their characteristics have made the comparison of different findings fairly challenging. For instance, parents of varied ethnicities tend to report some differences in terms of their engagement in childcare (Huntsinger and Jose, 2009). Similarly, parental education and family income have been demonstrated to have a significant impact on parental involvement (Kohl et al., 2000; Cano et al., 2019). Therefore, it should be stressed that neglecting cultural and demographic characteristics when measuring paternal involvement may bias the reported results. It is noteworthy that not all the studies (i.e., only 17 out of 27 studies) considered paternal involvement as a main focus of the research, by putting forward specific aims or hypotheses in the research questions. On the other hand, other studies merely measured the level of paternal involvement as a characteristic of the sample. Thus, it seems that parental involvement has often been regarded as a control measure rather than a variable of interest, despite playing an important role in the context of parental functioning. For this reason, great effort should be made to investigate the contribution of paternal involvement in childcare by testing specific research questions and hypotheses.

### Measurement of Paternal Involvement

The variety of conceptualizations and different approaches for assessing the complex construct of paternal involvement have led to an overall methodological inconsistency across studies. To address this major issue, a quality rating of measures has been proposed. According to the evaluation, a minority of studies included in this review used less appropriate methodological approaches to measure paternal involvement, especially in the context of behavioral studies (i.e., only 4 studies out of 16). However, some measures have not been considered appropriate as they were explicitly developed for assessing other constructs instead of parental involvement. In this case, we argued that these assessments could not be regarded as good practices, since the psychometric properties of a specific measure define its construct validity. For instance, two behavioral studies (Grossmann et al., 2002; Knauer et al., 2019) reported the amount of paternal involvement using a composite score, resulting from the combination of various and distinct aspects related to the broader concept of paternal role. Similarly, based on a semi-structured interview on parental quality, Carone et al. (2020) coded paternal investment using a composite scale which described both paternal beliefs regarding the importance of being a parent and the clear commitment to parenting. In such cases, the combination of different characteristics may have reduced the conceptual construct distinctiveness, limiting the understanding of the role of paternal involvement in childcare. Despite being classified as overall “good” measures, other studies could not stand up to criticisms in their methodology, especially those adopting a binary categorization of involvement (i.e., primary vs. secondary caregiver) based on a self-reported assessment. Therefore, the practice of dichotomization of the construct (Malmberg et al., 2007, 2016; Feugé et al., 2020), which has sometimes occurred in those studies thoroughly addressing caregivers' involvement (i.e., Abraham et al., 2014), could have resulted in an overall decrease of the measure sensitivity.

On the other hand, a larger proportion of neurobiological studies (i.e., 6 out of 11) adopted “fairly good” measures. In this vein, a joint couple interview (Kuo et al., 2016) has been considered as a questionable methodological practice to assess the involvement of caregivers, since it consisted of asking for both husbands and wives' agreement during a single home visit. In this setting, the presence of one member of the couple may have influenced the answers of the other member, thus producing an outcome measure based on biased processes. Additionally, measurements relying on few items have been rated as “fairly good” for not measuring the complexity of parental involvement (Feldman et al., 2010; Gettler et al., 2013; Nishitani et al., 2017), given that they hardly captured all the variability associated with the construct. Moreover, a limited methodological clarity has been outlined in those studies in which the amount of time spent in childcare was reported without a further specification of the measurements used to compute the scores (Wittfoth-Schardt et al., 2012; Waller et al., 2015). Moving to the measures classified as “good,” some neurobiological studies included the assessment of paternal involvement through standardized scales (Mascaro et al., 2013, 2014; Kuo et al., 2018). Mascaro et al. (2013) obtained caregiving scores from fathers and mothers separately, and then they computed the level of agreement between the ratings. This methodological approach could be considered far more suitable when compared to joint couple interviews, as it could generally prevent fathers' caregiving scores from being subject to the social desirability bias. Proving that both scores from mothers and fathers had good internal reliability, authors eventually decided to use maternal ratings in all analyses (Mascaro et al., 2013), since this has been described as a defensible practice (Cano et al., 2019). Other studies assessed paternal involvement by using a detailed set of items drawn from previous large-scale surveys, questionnaires, or studies (Feldman, 2000; Gettler et al., 2011; Abraham et al., 2014). On this note, Abraham et al. (2014) adopted a 30-question structured interview to determine the paternal active role in childcare. Specifically, this group of questions covered multiple caregiving domains, including parental responsibilities (e.g., take the child to the doctor), nurturing (e.g., change the diaper, prepare the bottle) and playful behaviors (e.g., tickle the child, blow on his/her belly). In this regard, different activity contents properly captured different nuances of parental involvement. Therefore, the thoroughness and multidimensionality of this measure could be considered as an optimum point of reference for conducting methodologically sound research addressing the contribution of paternal involvement to parenting, child and family outcomes.

### Associations Between Paternal Involvement and Sensitive Behaviors or Neurobiological Responses

Overall, only a limited number of studies included in this review found a significant link between paternal involvement and neurobiological responses toward infant cues or sensitive behaviors in fathers. As thoroughly reported, it is essential to consider that this finding may be related to the huge variability of the studies considered and the measures used for the assessment of paternal involvement. As an additional remark, the effects of some potential confounding variables such as the income, socioeconomic status (SES), education, distance from the family or emigration status (i.e., father lives in a different country), and other factors such as family responsibilities or stress have not been detected. Thus, the potential influence of these factors on paternal involvement, paternal sensitivity and neurobiological activations has not been explored due to the design of this study.

In the first place, two out of three behavioral studies (Grossmann et al., 2002; Brown et al., 2012) documenting a significant association between involvement and sensitive responsiveness showed some limitations that should be acknowledged. Specifically, Grossmann et al. (2002) found that paternal sensitive play at 24 months was associated with the presence of fathers at birth, but it was not correlated with a global measure of paternal involvement in childcare across the early postnatal period. In addition, the study by Brown et al. (2012) revealed a significant link between the two variables only at the second time point (i.e., 3 years), since no effect was found at 13 months. Among the behavioral studies included, the study by Feldman (2000) may be regarded as an encouraging result pointing to the importance of father involvement for paternal sensitivity. Importantly, these findings outlined that various aspects of paternal involvement added meaningfully to the prediction of the sensitivity of both mothers and fathers, highlighting the relevance of studying paternal involvement by adopting a dual co-parenting perspective. In terms of the methodological approach, it is of note that the study adopted a fine assessment for measuring paternal participation in childcare, for which both parents' perspectives were considered. Although other studies did not report significant associations between involvement and sensitivity, they found associations between paternal involvement and different domains related to parenting behaviors. For instance, Lewis et al. (2009) highlighted that primary caregiving fathers and their infants showed more positive emotional tone in the context of parent-child interaction compared to non-primary caregivers. Similarly, primary caregiving fathers also exhibited higher levels of positive mood than their spouses (Malmberg et al., 2007) and fostering a higher cognitive growth (Boechler et al., 2003) during father-child interactions. Moreover, it has been found that child attachment security was predicted not only by sensitive interactive behaviors but also by the degree of parental involvement (Fuertes et al., 2016; Brown et al., 2018). Interestingly, paternal sensitivity moderated the relation between involvement and child attachment security, showing a significant effect only when fathers were relatively less sensitive. Even though it has been considered here among the neurobiological studies, it should be noted that Abraham et al. (2014) reported an encouraging association between involvement and infant-parent synchrony during interactions, with primary-caregiving fathers and mothers showing greater synchrony with their own children than secondary-caregiving fathers.

Consistently, results from neurobiological studies stressed the importance of investigating the relationship between neurobiological responses toward infant cues and fathers' involvement in childcare by using proper measures. In this context, promising evidence has been outlined regarding the relationships between paternal involvement and the regulation of Cortisol and Prolactin (Gettler et al., 2011; Kuo et al., 2018). Specifically, increases in Cortisol after interacting with newborns predicted later paternal involvement in indirect childcare and play (Kuo et al., 2018). On the other hand, fathers engaging more with routine child care showed a greater short-term decline in Prolactin after playing with their children (Gettler et al., 2011). Given that the relationship between Prolactin and the motivation to approach and initiate paternal care has not been clarified (Storey et al., 2020), this evidence may provide a fruitful framework increasing our knowledge on the role of Prolactin in paternal behaviors. Regarding the level of Cortisol, contrasting results in literature highlight the need to rule out the possibility that an increase of this hormone in the dyadic context may depend on potential confounding factors, such as the time of sampling (Storey et al., 2020). Referring to the same study (Kuo et al., 2018), it may also be the case that high levels of Cortisol reflect the father's engagement with the new paternal role just after the baby birth, but it could be associated with less sensitive care later (Bos et al., 2018). With respect to the neural activations, relevant findings have been highlighted about the role of caregiving involvement in modulating fathers' neural responses to visual or auditory infant stimuli (Mascaro et al., 2013, 2014; Abraham et al., 2014). Building on the Life History Theory (Mascaro et al., 2013), a greater activity in the Ventral Tegmental Area and a moderate level of activation of the Anterior Insula were associated with higher paternal involvement and responsibility scores (Mascaro et al., 2013, 2014). Accordingly, a non-linear relationship between the activity of Anterior Insula and paternal engagement may suggest that an optimal level of arousal is linked to engaged parenting (Mascaro et al., 2014). Moreover, Abraham et al. (2014), demonstrated that assuming the role of a committed parent and engaging in active care of the offspring may modulate the neural responses that parents display toward infant cues, and it may trigger a global caregiving network. In fact, whilst mothers usually show higher subcortical activations and fathers greater activations in cortical socio-cognitive circuits, brain adaptability to the degree of involvement in childcare may involve the coactivation of these two networks (i.e., Amygdala, Superior Temporal Sulcus) in primary-caregiving fathers (Abraham et al., 2014). Importantly, the relevance of these findings could be ascribable to the quality of the measure used for the assessment, which may have captured in a detailed and reliable manner the level of paternal involvement in childcare.

Taken together, our findings suggest that the associations with paternal involvement appear clearer when addressing brain and hormonal responsiveness to infants rather than parenting sensitive behaviors. In this regard, consistent evidence has been outlined regarding the modulating role of paternal involvement on both neural and hormonal responses to infant stimuli. A possible explanation is that the contribution of paternal involvement to parental sensitivity at behavioral level may be more difficult to detect, since it could be moderated or mediated by other parent and child factors which have been associated with parenting sensitive behaviors (e.g., parental mental health, attachment state of minds and infant temperament) (Pelchat et al., 2003; Ewing et al., 2019). However, very promising results have also emerged from behavioral studies (Feldman, 2000), thus encouraging further research addressing the influence of father involvement on paternal sensitivity by using a fine methodological approach, and taking into consideration a dual perspective including both the members of the couple.

Overall, although some aspects related to parenthood (i.e., brain activations, hormonal pathways) may be hard-wired and biologically determined, these findings seem to corroborate the hypothesis that the activation of some Parental Brain circuits (Swain, 2011; Swain et al., 2014) might be adaptable to experiences and direct commitment in childcare (Provenzi et al., 2021). In this line, we suggest that further research on gay parents could potentially unravel the influence of caregiving experiences on different aspects of neural responsiveness related to parenthood, by ruling out potential confounding factors linked to biological factors and gendered parenting behaviors. For these reasons, studies on these families could provide less biased results for parental involvement, thereby reducing consistent differences between mothers and fathers ascribable to socio-cultural factors (Pleck, 2010). Furthermore, the initial evidence on the association between paternal involvement and sensitivity suggests the benefits also for heterosexual fathers to play a primary role as helpers in childrearing, thus eventually leading to a more egalitarian way of the division of childcare in those families. Paternal sensitivity aside, their engagement with childcare could therefore be related to other positive characteristics of father-infant relationships (e.g., positive emotional tone in the interactions) (Lewis et al., 2009), and it could have a positive effect on the child attachment security (Fuertes et al., 2016; Brown et al., 2018). Moreover, although the construct of involvement has been often applied exclusively to fathers, a higher level of engagement cannot be assumed a priori in mothers. Thus, according to a dyadic approach, in order to consider the interplay and balance of maternal and paternal care to understand the role of involvement in the family context, it is essential to measure the engagement with childcare in both members of the couple. Further studies should include the same construct and methodology for mothers and fathers in order to allow comparison between parents (Fagan et al., 2014).

## Limitations

It should be noted that this study shows some limitations. Regarding the neuroendocrine bases of fathering, few results have been discussed here about the modulation of Testosterone. As opposed to the large number of studies addressing the regulation of this hormone in fathers (e.g., Grebe et al., 2019; Storey et al., 2020), we did not find some consistent evidence from more complex situations requiring paternal sensitive responsiveness and commitment in child rearing. However, this aspect will need to be uncovered in future research, since some nuances to the relationship between testosterone level and parental care have been preliminarily suggested (Kuo et al., 2018; Corpuz et al., 2021). Importantly, the heterogeneity of the included studies and the measures used for the assessment of paternal involvement may have influenced the results obtained. For this reason, the appreciation of findings in the context of a careful description of each study is warranted (Provenzi et al., 2021). In the first place, it should be acknowledged that the predictive role of some confounding factors (e.g., demographic, cultural characteristics) on both paternal sensitive behaviors and neurobiological activations is hard to determine in our work. For this reason, we strongly suggest interpreting our findings in the light of potential confounding effects. Furthermore, the circumscribed assessment of selected components of fathers' committed behaviors (e.g., frequency of dyadic interactions) may have not captured the dynamics and the quality of father-child relationships across time, thus missing the relationship between paternal involvement and paternal sensitive behaviors. On this note, we suggest that a broader and more integrated understanding of paternal involvement would shed further light on multiple mechanisms related to parenthood, so this construct might be a more robust predictor (Palkovitz, 2019). A recent call for increased attention to the quality of fathers involvement may be of primary importance in this regard, thereby recognizing the importance of a multidimensional assessment of the construct (Schoppe-Sullivan et al., 2004). Although there doesn't seem to be an univocal approach to measuring the complex construct of father involvement, a clearer conceptualization and operationalization would consistently be a considerable objective for further research and meta-analyses (Rodrigues et al., 2021).

## Conclusions

This review has employed a systematic approach to identify the relationship between involvement in childcare and neurobiological responses to infant cues and sensitive behaviors in fathers. To date, only few studies have addressed the actual role played by paternal involvement in relation to these aspects. However, it has been suggested that the amount of invested caregiving may be related to the neural activations, fluctuations in hormonal levels, and behavioral characteristics of fathers when responding to infants. When compared to behavioral results, a more consistent association has been outlined between the paternal involvement in childcare and brain or hormonal responses to infant stimuli. To further understand the larger stability of this association, a greater theoretical understanding of father involvement as well as an appropriate and consistent assessment is recommended. On this note, promising results could be achieved also for behavioral studies when a fine methodology is approached. Additionally, future study addressing the paternal involvement in same-sex families would remarkably contribute to the literature, by ruling out possible confounding variables as the traditional gendered division of childcare. Eventually, paternal involvement may be considered as an active contributor to infants' wellbeing by improving the quality of the perinatal environment, thus consequently leading to some positive epigenetic consequences for the offspring (Linnér and Almgren, 2020). In line with our findings, engaging with their children might be additionally linked to sensitive behaviors and neurobiological responsiveness from fathers, thus ultimately supporting the quality of the relationships within families. In conclusion, this work includes a cohesive idea of how brain activity, hormones, behaviors can systematically be related to one another in the same study. Thus, it moves the potential of more researchers doing so, since this approach may provide a deeper and comprehensive understanding of paternal psychobiology.

## Data Availability Statement

The original contributions presented in the study are included in the article/Supplementary Material, further inquiries can be directed to the corresponding author.

## Author Contributions

MGi and SD conceived the study. MGi and MGe screened the articles, performed data-analysis, and wrote the manuscript. SD, PR, and PV critically revised the manuscript. MGi, MGe, PR, PV, and SD had access to all the data included in this systematic review and approved the submitted version.

## Funding

This study was funded by the Italian Ministry of Education, Universities and Research (MIUR) as part of the Departments of Excellence Initiative.

## Conflict of Interest

The authors declare that the research was conducted in the absence of any commercial or financial relationships that could be construed as a potential conflict of interest.

## Publisher's Note

All claims expressed in this article are solely those of the authors and do not necessarily represent those of their affiliated organizations, or those of the publisher, the editors and the reviewers. Any product that may be evaluated in this article, or claim that may be made by its manufacturer, is not guaranteed or endorsed by the publisher.
