# Supplementary material for: The Role of Paternal Involvement on Behavioral Sensitive Responses and Neurobiological Activations in Fathers: A Systematic Review
Source: Front Behav Neurosci. 2022 Mar 9;16:820884. doi: 10.3389/fnbeh.2022.820884 (PMC8959913; doi:10.3389/fnbeh.2022.820884)
Supplement: Supplementary file 1 [file Table_1.docx]

**Table S1.** Assessment of the risk of bias of behavioral studies using the MMAT or Mixed Methods Appraisal Tool (Hong et al., 2018)

| **Authors** | **Study title** | **Screening questions (for all types of studies)** | | | **Category of study designs: Quantitative nonrandomized** | | | | |
| --- | --- | --- | --- | --- | --- | --- | --- | --- | --- |
|  |  | S1. Are there clear research questions? | | S2. Do the collected data allow to address the research questions? | 3.1. Are the participants representative of the target population? | 3.2. Are measurements appropriate regarding both the outcome and intervention (or exposure)? | 3.3. Are there complete outcome data? | 3.4. Are the confounders accounted for in the design and analysis? | 3.5. During the study period, is the intervention administered (or exposure occurred) as intended? |
| Boechler et al., 2003 | *Father-Child Teaching Interactions: The Relationship to Father Involvement in Caregiving* | YES | YES | | CAN'T TELL | YES | YES | NO | YES |
| Brown et al., 2018 | *Associations between father involvement and father–child attachment security: Variations based on timing and type of involvement* | YES | YES | | YES | YES | YES | YES | YES |
| Brown et al., 2012 | *Father Involvement, Paternal Sensitivity, and Father-Child  Attachment Security in the First Three Years* | YES | YES | | YES | YES | NO | YES | YES |
| Carone et al., 2020 | *Gay and Heterosexual Single Father Families Created by Surrogacy: Father–Child Relationships, Parenting Quality, and Children’s Psychological Adjustment* | YES | YES | | YES | YES | YES | YES | YES |
| Feldman, 2000 | *Parents' convergence on sharing and marital satisfaction, father involvement, and parent–child relationship at the transition to parenthood* | YES | YES | | YES | YES | YES | YES | YES |
| Feugé et al., 2020 | *Adoptive gay fathers’ sensitivity and child attachment and behavior problems* | YES | YES | | YES | YES | YES | YES | YES |
| Fuertes et al., 2016 | *The effects of parental sensitivity and involvement in caregiving on mother–infant and father–infant attachment in a Portuguese sample* | YES | YES | | YES | YES | YES | YES | YES |
| Grossmann et al., 2002 | *The Uniqueness of the Child–Father Attachment Relationship: Fathers’ Sensitive and Challenging Play as a Pivotal Variable in a 16-year Longitudinal Study* | YES | YES | | NO | YES | CAN'T TELL | NO | YES |
| Kazura, 2000 | *Fathers' Qualitative and Quantitative Involvement: An Investigation of Attachment, Play, and Social Interactions* | YES | YES | | YES | YES | YES | NO | YES |
| Knauer et al., 2019 | *Parenting quality at two developmental periods in early childhood and their association with child development* | YES | YES | | YES | YES | NO | YES | YES |
| Laflamme et al., 2002 | *A Comparison of Fathers’ and mothers’ Involvement in Childcare and Stimulation Behaviors During Free-Play With Their Infants at 9 and 15 Months* | NO** | YES | | YES | YES | YES | NO | YES |
| Lewis et al., 2008 | *A comparison of father–infant interaction between primary and non-primary care giving fathers* | YES | YES | | YES | YES | YES | YES | YES |
| Lundy, 2002 | *Paternal socio-psychological factors and infant attachment: The mediating role of synchrony in father–infant interactions* | YES | YES | | NO | YES | YES | YES | YES |
| Malmberg et al., 2016 | *The influence of mothers’ and fathers’ sensitivity in the first year of life on children’s cognitive outcomes at 18 and 36months* | YES | YES | | YES | YES | YES | YES | YES |
| Malmberg et al., 2007 | *Parent–infant interaction: A growth model approach* | YES | YES | | YES | YES | YES | YES | YES |
| NICHD, 2000 | *Factors associated with fathers' caregiving activities and sensitivity with young children* | YES | YES | | YES | YES | NO | YES | YES |

**Table S2.** Assessment of the risk of bias of neurobiological studies using the MMAT or Mixed Methods Appraisal Tool (Hong et al., 2018)

| **Authors** | **Study title** | **Screening questions (for all types of studies)** | | | **Category of study designs: Quantitative nonrandomized** | | | | |
| --- | --- | --- | --- | --- | --- | --- | --- | --- | --- |
|  |  | S1. Are there clear research questions? | | S2. Do the collected data allow to address the research questions? | 3.1. Are the participants representative of the target population? | 3.2. Are measurements appropriate regarding both the outcome and intervention (or exposure)? | 3.3. Are there complete outcome data? | 3.4. Are the confounders accounted for in the design and analysis? | 3.5. During the study period, is the intervention administered (or exposure occurred) as intended? |
| Abraham et al., 2014 | *Father’s brain is sensitive to childcare experiences* | YES | | YES | YES | YES | YES | YES | YES |
| Feldman et al., 2010 | *Natural variations in maternal and paternal care are associated with systematic changes in oxytocin following parent–infant contact* | YES | | YES | YES | YES | CAN'T TELL | YES | YES |
| Gettler et al., 2013 | *Progesterone and estrogen responsiveness to father-toddler interaction* | YES | | YES | NO | YES | YES | YES | YES |
| Gettler et al., 2011 | *Short-term changes in fathers' hormones during father–child play: Impacts of paternal attitudes and experience* | YES | | YES | NO | YES | YES | YES | YES |
| Kuo et al., 2018 | *Fathers’ cortisol and testosterone in the days around infants’ births predict later paternal involvement* | YES | | YES | YES | YES | NO | YES | YES |
| Kuo et al., 2016 | *Individual variation in fathers’ testosterone reactivity to infant distress predicts parenting behaviors with their 1-year-old infants* | YES | | YES | YES | YES | NO | YES | YES |
| Mascaro et al., 2014 | *Behavioral and genetic correlates of the neural response to infant crying among human fathers* | YES | | YES | YES | YES | YES | CAN'T TELL | YES |
| Mascaro et al., 2013 | *Testicular volume is inversely correlated with nurturing-related brain activity in human fathers* | YES | | YES | YES | YES | NO | YES | YES |
| Nishitani et al., 2017 | *Genetic variants in oxytocin receptor and arginine-vasopressin receptor 1A are associated with the neural correlates of maternal and paternal affection towards their child* | YES | | YES | YES | YES | YES | YES | YES |
|  | | | | | **Category of study designs: Quantitative randomized** | | | | |
|  |  |  |  |  | 2.2. Are the groups comparable at baseline? | 2.3. Are there complete outcome data? | 2.4. Are outcome assessors blinded to the intervention provided? | 2.5. Did the participants adhere to the assigned intervention? | 2.2. Are the groups comparable at baseline? |
| Waller et al., 2015 | *Attachment representation modulates oxytocin effects on the processing of own child faces in fathers* | YES | YES | | YES | YES | YES | YES | YES |
| Wittfoth-Schardt et al., 2012 | *Oxytocin Modulates Neural Reactivity to Children’s Faces as a Function of Social Salience* | YES | YES | | YES | YES | NO | YES | YES |
